# SENSITIVITY OF FALL RISK CUTOFFS USING LAB AND REMOTE MEASURES ON OLDER PROSTATE CANCER SURVIVORS

**DOI:** 10.1093/geroni/igae098.2613

**Published:** 2024-12-31

**Authors:** Carolyn Guidarelli, Sydnee Stoyles, Nathan Dieckmann, Alexandra Sokolova, Julie Graff, Eric Roeland, Kerri Winters-Stone

**Affiliations:** Oregon Health & Science University, Portland, Oregon, United States; Oregon Health & Science University, Portland, Oregon, United States; Oregon Health & Science University, Portland, Oregon, United States; Oregon Health & Science University, Portland, Oregon, United States; Oregon Health & Science University, Portland, Oregon, United States; Oregon Health & Science University, Portland, Oregon, United States; Oregon Health & Science University, Portland, Oregon, United States

## Abstract

Prostate cancer survivors (PCS) treated with androgen deprivation therapy (ADT) have increased fall risk due to treatment-related side effects on musculoskeletal health. Fall risk is typically measured in the lab or clinic using objective assessments validated in community-dwelling older adults. It is unknown whether the same cutoffs will identify PCS at risk of falls. Conducting these assessments remotely has become more prevalent in research; however, it remains unclear whether different cutoffs are required using this modality. Thus, we explored the sensitivity and specificity of traditional fall risk cutoff scores using lab and remote measures to correctly classify faller status among older PCS. We used baseline data on 243 older (mean age 74.5 years) PCS from the GET FIT Prostate trial (NCT03741335). We captured fall history over the past year and completed lab and/or remote assessments of the 3-meter Timed Up and Go (TUG), 4-meter usual walk (4mUW), 5x Sit-to-Stand (5xSTS), and short physical performance battery (sPPB). We classified fall risk as TUG ≥13.5s, 4mUW ≤1.0m/s, 5xSTS ≥12s, sPPB ≤9, and faller ≥1 fall. Overall, 37.9% (n=92) reported a fall within the last year. Each test had similar sensitivity and specificity with AUCs of 58.5%, 56.3%, 55.4% and 56.0% for the lab-based TUG, 4mUW, 5xSTS, and sPPB, respectively. AUCs for remote testing were similar. Regardless of measure or modality, traditional fall risk cutoff scores do not accurately classify faller status in PCS exposed to ADT, leaving clinical practice with few tools to identify men at risk of falls.

